# A Geospatial Analysis of Access to Ethnic Food Retailers in Two Michigan Cities: Investigating the Importance of Outlet Type within Active Travel Neighborhoods

**DOI:** 10.3390/ijerph17010166

**Published:** 2019-12-25

**Authors:** Greg Rybarczyk, Dorceta Taylor, Shannon Brines, Richard Wetzel

**Affiliations:** 1 Department of Geography, Planning and Environment, University of Michigan-Flint, Flint, MI 48502, USA; dick.wetzel@gmail.com; 2 The Michigan Institute for Data Science (MIDAS), Ann Arbor, MI 48108, USA; 3 The Centre for Urban Design and Mental Health, London SW9 7QF, UK; 4 School for Environment and Sustainability, University of Michigan, Ann Arbor, MI 48108, USA; dorceta@umich.edu (D.T.); sjbrines@umich.edu (S.B.)

**Keywords:** food access, ethnic food, service area analysis, GIS, urban design, GWR, local regression, space syntax, demographic characteristics

## Abstract

To date, the research that examines food accessibility has tended to ignore ethnic food outlets. This void leaves us with a limited understanding of how such food stores may, or may not, impact food security. The study discussed herein addressed this by conducting a geospatial assessment of ethnic food outlet accessibility in two U.S. cities: Flint and Grand Rapids, Michigan. We used Geographic Information Systems (GIS) tools to create a revealed accessibility index for each food outlet, and used the index to determine access within active travel service areas. We utilized an ordinary least squares regression (OLS), and two local models: spatial autoregression (SAR) and geographically weighted regression (GWR) to enhance our understanding of global and localized relationships between outlet accessibility and type (while controlling for known covariates). The results show that the local models outperformed (*R*^2^ max = 0.938) the OLS model. The study found that there was reduced access to ethnic restaurants in all service areas of Grand Rapids. However, in Flint, we observed this association in the bicycling areas only. Also notable were the influences that demographic characteristics had on access in each city. Ultimately, the findings tell us that nuanced planning and policy approaches are needed in order to promote greater access to ethnic food outlets and reduce overall food insecurity.

## 1. Introduction

Food insecurity is a growing concern in the U.S. According to the U.S. Department of Agriculture, 11.1%, or roughly 37.2 million people, lived in households that were food insecure. Being food insecure means that such households are unsure of having, or are unable to, obtain enough food to meet their needs because they lack money, transportation, or other resources to acquire adequate food [1]. To make matters worse, many urban areas that once contained a multitude of healthy food outlets now have no or few places in which residents can purchase healthy foods. Instead, purveyors of junk foods have supplanted healthy food vendors. Two unfortunate outcomes of this transition have been that some urban residents now travel further to obtain healthy food, or they patronize smaller convenience stores, grocers, or restaurants that may not contain many healthy food options [2,3,4].

Current research on the role of small food outlets in mitigating food insecurity is limited. The majority of research on small food vendors is focused on the traditional corner store, gas stations and liquor stores—places that have been found to stock a limited amount of healthy foods [5]. Small food outlets are essential in the food landscape. They are a convenient source of food for many people. However, some researchers find that residents who obtain most of their food from these vendors are at risk for health problems such as obesity [6]. Researchers have also found that food sold in the smaller stores are more expensive than food that is sold in supermarkets and full-line grocery stores. Scholars also report that small food outlets tend to sell lower quality food than supermarkets and grocery stores [7,8,9].

In recent years, a growing number of researchers, public health practitioners, and food advocates have expanded their research to include ethnic food stores. There is interest in assessing the potential for neighborhood ethnic markets to enhance food access [10,11,12,13]. Ethnic food stores are significant because they often serve ethnic enclaves, low-income, and immigrant communities with culturally desired foods that help people to meet their nutritional requirements. In places such as Brooklyn, bodegas–which typically has about 400 square feet of vending space-comprise more than 80% of the food retailers. That is, in such urban areas, residents purchase food from ethnic retailers. This is particularly true for those who do not have private transportation to go to distant grocery stores [2,10,14].

Recognizing the vital niche that they serve, efforts are underway in some cities to assist ethnic food stores to sell more healthy foods. For example, New York City, which has over 10,000 bodegas piloted the Healthy Bodegas Initiative in 2005 [14]. The program demonstrated that, with efficient programming, store owners increased their healthy food offerings within many of the bodegas in the city [15]. Khojasteh and Raja [13] found that Middle-Eastern ethnic food stores in Buffalo, New York, sold a variety of healthy foods while Short et al. [16] report that ethnic food retailers sold food at reduced costs. Gittelsohn et al., [17] argue that ethnic grocers help to alleviate food insecurity while promoting positive neighborhood change. Considering that by 2050, nearly half of U.S. food consumers will be ethnic minorities, we must pay more attention to ethnic food outlets [18].

Examining the prevalence of ethnic food outlets in two cities helps us to understand how the presence of such food vendors is related to the mode of travel and other factors that influence food access [19]. This approach is supported by past studies that reveal that neighborhood residents use various means of travel to obtain food. Clifton [20] discovered that low-income food shoppers in Austin, TX, utilized a multitude of travel modes—including transit, borrowing vehicles, or ride-sharing—to reach their destinations. While a growing number of food access studies have incorporated different mobility options [21], a common failing has been the way neighborhood is conceptualized. For instance, many researchers have viewed the density of food outlets as an indicator of food access [22,23], while others create buffers around Census units and assess the type of food outlets located within these geographic areas [24]. Unfortunately, these approaches do not fully account for the edge effect. That is, residents living on the edge of the neighborhood are likely travel beyond the neighborhood boundary for food. Hence, food stores located outside of a given neighborhood also serve residents in a study area. While we build on earlier studies in this genre [25,26], the current study proposes a service area approach combined with geospatial analysis to examine ethnic food access. The research had three objectives. First, use exploratory spatial data analysis (ESDA) to visualize the geographical distribution of ethnic food outlets in two Michigan cities. Second, implement a revealed accessibility index to compare ethnic food access. Lastly, adopt a geospatial modeling approach to assess the relationship between the location of ethnic food outlets and outlet type within three active transportation service areas. 

## 2. Materials and Methods

### 2.1. Study Area and Data

**Figure 1:**
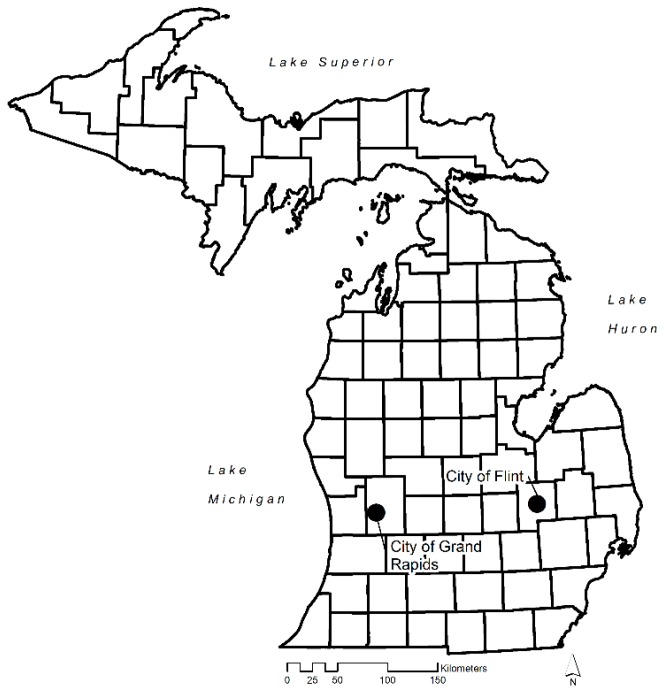
The State of Michigan and our two study cities: Flint and Grand Rapids.

The study areas used in this research are two cities in Michigan–Grand Rapids and Flint (Figure 1). Grand Rapids is the second-largest city in Michigan; it has been described as one of the fastest growing cities in the U.S. [27]. Since 2010 the population has grown by 6.5%; it was 200,217 in 2018. Grand Rapids is a racially heterogeneous city. Whites comprise 59.7% of the city’s population, African Americans make up 19.9%, LatinX constitute 15.3%, Asians account for 2.1%, and Native Americans 0.4% of the population [28].

In contrast, Flint is characterized by severe population losses and a precipitous economic decline since the 1970s. The shrinking of the automobile industry and increasing suburbanization has resulted in a 50% population loss since 1960 [29,30]. According to the Census, Flint’s population has by 6.1% since 2010. The city’s 2018 population was 95,943. Whites make up 37.5% of Flint’s population, African Americans account for 53.9%, LatinX make up 3.9%, Native Americans comprise 0.6%, and Asians 0.4% of the population [28]. Many of Flint’s residents live in neighborhoods with low food access: approximately 73% of Flint’s residents live at least 1000 m from a food source in 2009 [31]. Table 1 presents other statistics that compare the study areas.

### 2.2. Data

**Figure 2:**
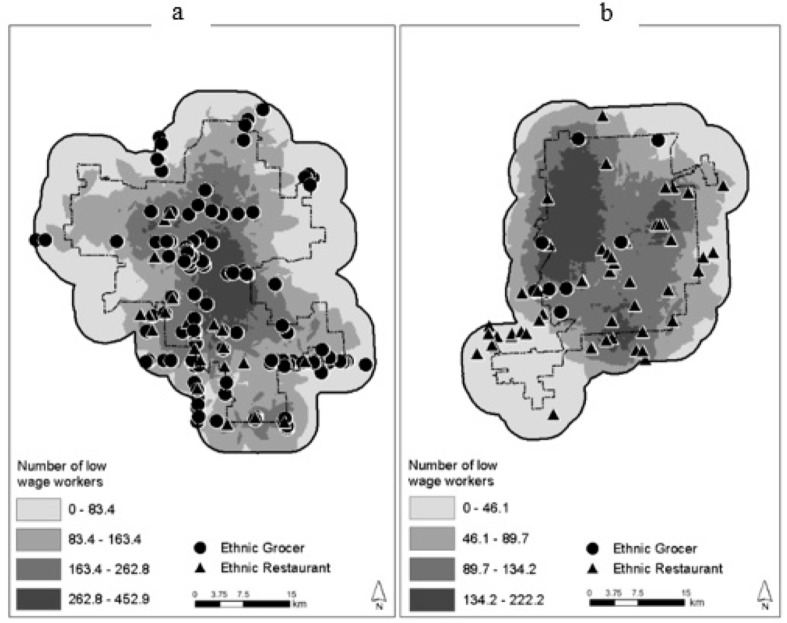
Spatial patterning of ethnic food outlet locations and density of low-wage employees within the study sites: Grand Rapids (**a**) and Flint (**b**), Michigan.

All covariates were collected within each of the city limits and also within an area extending 4.0 km buffer beyond (see Figure 2). We chose this as the overall study area to minimize edge-effects and account for the diversity of factors, including food outlets, outside the city boundary. The food outlet database was created in 2012 and data for the factors being analyzed were collected between 2010 and 2012. The covariates considered in this research were mostly obtained from government sources and pertain to well-known food access predictors: demographic characteristics, environment, and urban morphology.

#### 2.2.1. Ethnic Food Outlets

The food outlet database used in this research was compiled from the Reference USA (http://www.referenceusa.com/) and Orbis (https://www.bvdinfo.com/en-gb/our-products/data/international/orbis). We also obtained data on food venders from the Michigan Department of Agriculture. The data were collected from October to December 2012. The databases contain business information such as the company name, phone number, address, sales (dollars), owner’s name, ethnicity, building size, address, latitude/longitude coordinates, and Standard Industrial Classification (SIC) codes. We followed the protocol set forth by Taylor and Ard [32] to classify food outlets in our database. The SIC codes most commonly used in this research were: 5411 (convenience store, food retail), 5311 (retail shops), 5541 (service stations), 5431 (fruits, vegetables, produce), 5499 (Mexican, Latin, American food), and 5812 (restaurants).

We used the same subtype categorization methodology as Taylor and Ard [32] to identify the ethnic restaurants and grocers in our database. For the remainder of this paper, such food outlets will be classified simply as “restaurants” and “grocers.” We obtained the following information for the ethnic food retailers: company name, owner’s name, owner’s ethnicity, cuisine code, and sales revenue. First, we grouped major SIC codes into usable subtype categories by reviewing the “company name”, “owner’s name”, and owner’s ethnicity to help us determine if the owner had identified his or her ethnicity or if the food outlet sold ethnic food. As a result, we found these types of retailers–Asian, Cajun, Caribbean, Chinese, Cuban, English, French, German, Greek, Hawaiian, International, Italian, Jamaican, Japanese, Korea, Mediterranean, Mexican, Polish, Thai, and soul food. Next, we examined the cuisine code to see how the restaurants were labeled in the databases we drew our information from. The two cuisine codes used were ethnic and American. If the cuisine code was missing, we used the Internet to find pictures of the food establishment and looked at the signage and building advertising. For example, if the signage for a restaurant was written in a foreign language, that language occupied more than half of the sign, and the establishment served or sold ethnic foods, it was considered an ethnic food outlet. We also used Google Street View and Bing maps to help us categorize the restaurant. We used this same technique to identify ethnic grocery stores that were not labeled in Reference USA, Orbis, or the U.S. Department of Agriculture databases.

We also used information on annual sales to further refine our categorization. We used the sales categories outlined by the Food Marketing Institute (FMI) in our definitions (http://fmi.org/research-resources/supermarket-facts). The resulting ethnic food outlets were then plotted using the coordinates in GIS software (Environmental Systems Research Institute, version 10.5). The *ex*-*post facto* analysis included a manual geo-locational and outlet type verification process conducted by three research assistants. The final set of outlets used in this research are shown in Table 2.

#### 2.2.2. Socioeconomic, Environmental, and Urban Design Covariates

Food access, and hence diet, can be influenced by many demographic and environmental factors [33]. Therefore, we collected several demographic and environmental indicators from the United States (U.S.) Environmental Protection Agency’s (EPA) Smart Location Database (SLD), the U.S. Census Bureau, and other government sources. The SLD contains 90 different metrics reflecting well-known demographic and environmental indicators for the entire U.S; it is aggregated to the Census Block Group (CBG) level [34]. These factors were used to help us assess neighborhood quality. Hence, we obtained indicators for land-use diversity, network design, accessibility, demographics, and employment status. We also collected information from city, state, and federal sources on other quality-of-life indicators such as schools, parks, motorized and non-motorized transportation networks, land-use diversity, bicycle/pedestrian crashes, brownfields, and crime incidences.

Urban design has been shown to be an essential factor in food accessibility [35], yet it is not typically studied in food access research. Space syntax has long been a means to examine how the urban form influences human movement and cognition. The theoretical framework is based on the notion that accessibility is moderated by urban configurations and may not be solely dependent on the destination or attraction [36]. For instance, scholars report that longer lines of sight, reduced path angularity, and integrated or connected spaces–which is dictated by urban morphology-facilitate wayfinding and accessibility [37]. This has obvious implications for food acquisition, consequently, we incorporated urban design metrics into our study of food access. To carry this out, we utilized the freeware Depthmap software (Version X) developed by University College London (https://github.com/SpaceGroupUCL/depthmapX/).

### 2.3. Service Area Delineation and Data Aggregation

In this research, we created three active living (bicycling, mass-transit, and walking) service areas where the ethnic food outlet serves as the center of each zone. As mentioned earlier, this method captures the heterogeneity around each vendor. The method also minimizes biases that arise from edge effects (edge effect bias is a common challenge in food access research) [38]. We created the service areas by using the road network of each city, ArcGIS software, and the Network Analyst extension. The service area distances—based on definitions used in previous active travel research–were defined as 0.40 km to represent walking; 5.36 km and 0.40 km distance along each mass-transit route and around each stop, respectively; and 8.04 km to represent a feasible bicycling distance [26,39,40,41]. Network distances, instead of straight-line distances, were used because they represent the travel environment more accurately [42]. The exposure variable and all covariates were added to the service areas using areal interpolation. This method is commonly used to integrate spatial data from one set of zonal units with another. In this case, the source data is treated as a continuous density surface; the source data is joined to the target dataset then multiplied by the ratio of spatial overlap between the areal units [43]. All covariates were normalized by the service area (kilometer) to minimize errors associated with the modifiable areal unit problem (MAUP). The MAUP is another well-known problem that arises in accessibility studies [44].

### 2.4. Geovisualizations and Spatial Analysis

Our first objective (Objective A) in this study was to use ESDA to assess the spatiality of the ethnic food outlets in relation to the demographic characteristics of each city. To do this, we plotted each food outlet’s latitude and longitude in ArcGIS. We used kernel density estimation of the number of low-wage workers (a socioeconomic indicator) in the 2010 Census block groups. Kernel density estimation is often used to visualize the intensity of a phenomenon [43]. We used another ESDA technique, calculating the global Moran’s *I* index, to identify spatial autocorrelation (or clustering) of the reveal accessibility index (RAI) values in each city [45]. The index ranges in value from −1 to +1. The positive values indicate positive autocorrelation, and negative values highlight an inverse spatial relationship [46]. In addition to the Moran’s *I* index, *z*-scores were reported for statistical analyses. If the *z*-score values are at levels of ±1.96, the randomness test is rejected and the pattern is spatially correlated [47].

### 2.5. Developing the Outcome Variable: Revealed Accessibility Index

Our second objective (Objective B) was to create a RAI to represent food access for each restaurant and grocer in our active travel neighborhoods. Here, we build on the works of Miller and Shawn [48] and Lee et al. [49]. The index is unique because it accounts for food outlet usage (as indicated by reported revenue), square footage, quantity of other competing food outlets, and travel cost (i.e., distance decay within each neighborhood). A high index output is caused by low competition and high accessibility of each ethnic food outlet (EFO), while a low index indicates high competition and low accessibility. The index form is as follows:

(1) $$RAI=\;\left(\frac{1}{m-1}\right)\underset{k\neq j}{\sum }w_{k}c_{jk}^{-2}$$

where: m = all other food outlets; w = normalized EFO revenue (outlet structural sq. footage); $c_{jk}$ = impedance function based on network buffer distance to the −2 exponent.

The cost of −2 represents a regularly used power function that measures the decrease in spatial interaction between an origin and destination [50]. The decrease represents a distance decay function; in other words, a resident who lives further away from an EFO in the network zone would experience a greater a “cost” in reaching their food outlet destination. The usage (w) of each EFO was established by normalizing the revenue by the reported square footage of the structure. The building square footage was used because it contributes to store attraction; square footage also makes it possible to compare the size of all the EFO’s [51].

### 2.6. Data Preprocessing

From the initial database of all possible variables influencing associability, we adopted a two-pronged approach to determine the final set of covariates. We conducted a Pearson product-moment correlation analysis among the RAI and all pertinent covariates in each active living zone. Those which were statistically significant (*p*-value < 0.05) were retained in the model for further analysis. The correlation analysis was conducted using SPSS (IBM Inc., Version 22) statistical software. We also studied the variance inflation factor (VIF) to identify whether there was multicollinearity among the covariates. VIF values that are less than 10 are acceptable [52]. We did not find any evidence of multicollinearity among the covariates used in this study. The retained variables were consistent among each active travel zone in both cities for comparison purposes. The final covariates and their definitions are shown in Table 3.

### 2.7. Global and Local Model Development

The last goal (Objective C) was to develop a geospatial modeling approach to enhance our understanding of access to ethnic foods. We used a multivariate ordinary least squares (OLS) regression model to examine the global linear relationship between the outcome variables and ethnic food outlet type. The OLS model was considered the base model in this study. All variables were entered simultaneously and were considered fully adjusted. Because the OLS models violate the assumption of a normal distribution and uncorrelated error terms when spatial heterogeneity is present, we proposed a local modeling approach.

Local models can help to identify relationships that remain hidden by global models, such as OLS. Therefore, we created two local models to account for spatial autocorrelation among dependent or independent variables. The first one enlisted was a simultaneous spatial autoregressive (SAR) model. The SAR model is based on the assumption that the exposure variable is affected by surrounding values from other factors [53]. The model is also typically used when spatial autocorrelation is hypothesized to affect the exposure variable and covariates; it is also recommended when spill-over effects are possible [54,55]. Spatial relationships are modeled based on neighborhood distances between the exposure variable locales *i* and *j* and an *n x n* set of spatial weights. The model form is as follows:

(2) y=ρWy+Xβ+WXy+ ε

The *ρ* is the spatial lag parameter and *W* is a spatial weights matrix representing the proximity between each pair of EFO’s *i* and *j*, *β* is a K by 1 vector of regression coefficients *X*; WXγ denotes the autoregression coefficient (γ) of the spatially lagged covariates [56]. The variable *ε* is the vector of independent error terms, which may not be identically distributed [57]. We used the freeware SAM software to design this model [58].

To explore the spatial dimensions of access to ethnic foods more fully, we incorporated geographically weighted regression (GWR) into this research. The GWR approach fits a separate model for each EFO to help us detect nuanced influences on the RAI. The GWR model allows all regression variables to vary spatially within each zone of the two study cities. The method produces coefficients for each EFO and is particularly useful when there are many confounding variables and interaction effects that cannot be controlled for [59,60]. The spatially variant factors are treated with a weighting scheme based on distance and bandwidth. The weights (i.e., kernel function) are based on a distance-decay algorithm defined as the Gaussian distribution wherein greater salience is applied to variables that are closer to the EFO and less when further away. In this study, we chose the calibration method that minimizes the AICc of each regression model and the Gaussian adaptive bandwidth function. The GWR equation is elaborated on by Fotheringham et al. [61] and takes the form:

(3) $$y_{i}=\beta_{0}\left(u_{i\;},v_{i}\right)+\underset{k}{\sum }\beta_{k}\left(u_{i},v_{i}\right)x_{ik}\;+\varepsilon_{i}$$

where $\left(u_{i\;},v_{i}\right)$ denotes the coordinates of the exposure variable *y*; $\left(u_{i\;},v_{i}\right)$ denotes the coordinates of *i*; and $\beta_{0}$ and $\beta_{k}$ represent the local estimate intercept and influence of factor *k* at location *i*, respectively; and ε is the random error term which accounts for varying values across space [61,62]. The key characteristic of the GWR equation is that locations closer to *i* possess a stronger influence on the estimation of $\beta_{k}\left(u_{i},v_{i}\right)$ than locations further away. We utilized the freeware GWR4 software to conduct the GWR models [63].

The performance of each model (OLS, SAR, and GWR) used in this study was assessed by reviewing the model diagnostic tests: coefficient of determination (*R*^2^) and AICc. The latter is considered the preferred means of measuring model robustness. The best model is the one with an AICc index value of three less than any other model’s AICc index. In contrast, an elevated *R*^2^ index indicates a stronger model because the independent variables explain more of the variance in the dependent variable [61,64]. We also tested the standardized residuals from each model for spatial autocorrelation using the Global Moran’s *I* statistic. If the index is statistically significant the independence of observations assumption is violated [65].

## 3. Results

### 3.1. The Spatial Distribution of Ethnic Food Outlets and Demographic Characteristics

Regarding Objective A—which was to visualize the comparative differences between restaurants and grocers in each city using ESDA—Figure 2a displays a random pattern of both types of outlets in Grand Rapids. Low SES is concentrated in the center of the city and there appears to be several EFO’s in the core of the city. This pattern suggests that food insecurity among the city’s low-income residents may be low. In contrast, the spatial relationship between food outlets and low socioeconomic status in Flint indicates that there is reduced access to ethnic food outlets in the northwest section of the city (Figure 2b). However, global Moran’s *I* outcomes showed there was no significant clustering of food retailers (i.e., RAI) throughout space (Table 4).

### 3.2. Accessibility within Walking Service Areas

The model results obtained from the walking zone analysis are shown in Table 5. The diagnostic tests indicate that the models’ strengths were marginal in each city, except for Flint, where the SAR model’s *R*^2^ explained 51% of the variation using the selected covariates. The SAR model exhibited a 3.43% reduction in AICc when compared to the OLS model, suggesting a better fit. Similarly, we found the local GWR model had the strongest fit for Grand Rapids: the *R*^2^ index increased 64.28% from the OLS model’s value, indicating a better fit likely because spatial heterogeneity was captured.

The type of ethnic food outlets was significant in the assessment of walking areas around such stores in Grand Rapids. We found that ethnic groceries were more accessible than ethnic restaurants; see the statistically significant negative EFO coefficients in the OLS and SAR models (*p* < 0.05). It was also evident that environmental and urban morphology indicators had an effect on the accessibility of ethnic food outlets in Flint. The density of pedestrian infrastructures and the shortest line distance (shortest path) had a significant (*p* < 0.01) and positive influence on accessibility; while isovist area had a significant (*p* < 0.05) negative impact on accessibility.

### 3.3. Accessibility within Mass-Transit Service Areas

Table 6 presents the best-fitting global and local models for predicting ethnic food outlet accessibility in the mass-transit areas around each establishment. Collectively, the outputs were stronger than the walking area models. In Flint, the greatest amount of explained variance occurred with the GWR model (*R*^2^ = 0.93). We discovered a 5.86% increase in *R*^2^, and 63.34% decrease in AICc when compared to the OLS model; these results indicate a more robust model due to the accountability of spatial effects. The local SAR model was strongest in the Grand Rapids analysis. We found an increase of 10.35% in *R*^2^ and a reduction of 17.70% for the AICc value when contrasted with the OLS model.

The associations between type of ethnic food outlet and access was significant in Grand Rapids (*p* < 0.01). The negative coefficient indicates that groceries were more accessible than restaurants when zonal conditions were held constant. The association held true for the SAR and OLS models. The GWR coefficients suggest that the association varied spatially. Of the SES factors that influenced access, gender was most significant in both cities (*p* < 0.01). Both the local (SAR and GWR) and global models (OLS) in Flint and Grand Rapids indicated that males experienced reduced access to ethnic food stores when compared to females in the same neighborhood. Table 6 also shows that the density of multi-modal intersections had a positive, and statistically significant (max *p*-value = 0.01) impact on access in the mass-transit neighborhoods of each city. We also found that the shape of the environment had bearing on access to ethnic food vendors in Flint. The mean angularity of the pathways (road network radii) was significant (*p* < 0.01) and adversely affected access in Flint’s mass-transit zone.

### 3.4. Accessibility within Bicycling Service Areas

The results of the global and local models’ prediction of ethnic food access in bicycling zones are presented in Table 7. The strength of all the models were robust. In Flint, we found that the GWR model explained the greatest amount of variance (*R*^2^ = 0.852); there was a reduction in the explanatory power of AICc (0.428%) when it was when compared to the OLS model. The strongest model in Grand Rapids resulted from the SAR analysis (*R*^2^ = 0.530); this model exhibited a 6.41% increase in *R*^2^ and a 1.77% decrease in AICc when compared to the OLS model. The strength of the SAR model may be attributable to the model’s ability to account for spatial effects.

Ethnic food outlet type was important in predicting access in the bicycling zones in each city. There was a significant inverse association between ethnic restaurants and access in Grand Rapids (*p* < 0.01). That is, ethnic grocery stores were more accessible than restaurants in the city. This was true of Flint also. Race had significant impacts on access in both cities. That is, non-white residents had greater access to ethnic food outlets (*p* < 0.01) that white residents. The GWR coefficients in each city indicate that this relationship is spatially invariant. Other demographic coefficients also indicate that those with a high school diploma had marginally increased access to ethnic food outlets (*p* < 0.05) in Flint than residents who had not graduated from high school. Similarly, environmental and urban morphology were also important factors influencing access to ethnic food establishments in Flint. The density of parks (*p* < 0.05) and occluding radials (occlusivity) (*p* > 0.01) had a positive association with the outcome variable (RAI).

## 4. Discussion and Conclusions

Scholars have paid scant attention to ethnic food resources and their accessibility [10,11]. The majority of the studies investigating this phenomenon have relied on qualitative assessments [15] or empirical models that are not adjusted to accommodate spatially varying relationships [66]. Additionally, few have utilized a service area analysis to quantify the full range of factors affecting ethnic food access and control for edge-effects. The omission of robust empirical and geospatial approaches leaves us with an information void about access to ethnic foods. The current research set out to fill this gap by examining ethnic food access in two different cities. We found that low-wage workers living in the northwestern portion of Flint had low access to ethnic food outlets. We then demonstrated that ethnic restaurants were largely inaccessible in each active living area in Grand Rapids. The relationship held true in Flint, but only in the bicycling zone. The study demonstrates that measuring ethnic food access isn’t a simple task. Several statistically significant demographic, environmental, and urban design indicators influenced access in each city.

Our first research objective was to utilize ESDA to examine the spatiality of ethnic food outlets in each city and compare this to the density of low-wage workers. We found that the density of outlets, as well as their spatial patterning, differed in each city. In Flint, we observed clustering of outlets near the center of the city and as well as close to the perimeter. The juxtaposition suggests that food deprivation could be a problem in Flint. This observation has also been noted by Sadler et al. [67]. The results of this study should prompt further investigations into food access. Efforts should also be made to provide more opportunities for residents to gain access to healthy foods. We found a greater density and dispersion of ethnic food outlets in Grand Rapids. In the city’s central business district where low-income residents were present, there were ample ethnic food outlets.

The second objective in this research was to develop an accessibility measure (the RAI) that accounted for distance, outlet usage, and competing food resources in three active travel service areas. Our last objective was the implementation of a geospatial (local) model to detect associations between outlet type and the RAI. Even though the local model coefficients largely mirrored the results of the OLS model, the results from the latter proved more robust. The study also found that the type of ethnic food outlet matters—especially in Grand Rapids. In this city, we found that ethnic restaurants were not as accessible as ethnic grocery stores. Unlike past research which has found ethnic restaurants may help alleviate food insecurity and improve health outcomes [68], our study suggests that ethnic restaurants may not be an accessible food resource. Ethnic restaurant accessibility may be influenced by food prices, location, business hours, or lack of culturally appropriate foods [69]. Hence, more attention should be paid to the operation and role that ethnic restaurants can play in urban areas. Additionally, we recommend that planners consider incentives to prompt partnerships between local farmers and ethnic restaurants to facilitate more healthy food offerings [70]. Except from the bicycling service areas analysis, ethnic food outlet accessibility in Flint wasn’t as dependent on food outlet type as demographic characteristics, environmental, and urban morphological conditions within the active travel services areas.

In the walking areas surrounding each ethnic food outlet in Flint, the local models illustrate that environmental and urban morphology factors contributed to accessibility. The density of pedestrian-orientated intersections had a positive influence on access and the GWR coefficients indicated that this was more important in some areas than others. A positive relationship was also found between two urban form indicators—shortest line distance and isovist areas. Together, this association suggests that walkable areas surrounding the food outlets which contain a high number of linear pathways with strong sight lines may increase accessibility. These findings are corroborated by Turner et al. [71]. Similarly, urban form and environmental conditions in mass-transit areas were also found to affect ethnic food accessibility. We discovered a positive and spatially consistent relationship between areas with a high quantity of multi-modal intersections and outlet accessibility. This finding indicates that residents who rely on alternative transport may have greater accessibility to ethnic food outlets [72]. The finding also lends credence to past findings which suggest that procuring healthy foods often involves multiple travel modes [19]. We found that gender had a marginally significant impact on ethnic food access. The local model coefficients in Grand Rapids showed that males experienced less access to ethnic food outlets than females. Supporting results have been reported elsewhere [73]. The socio-economic conditions in each city’s bikeable service area also exerted influence on ethnic food outlet accessibility. In Flint, the relationship between access and race was strong; a similar finding was evident in Grand Rapids. Non-whites had greater access to ethnic food outlets than whites. This may be the case because non-whites find these outlets more accessible for the culturally desirable and reasonably priced foods. We also showed that the urban design was relevant in this zone. In Flint, we found a positive link between occlusivity and access: the increase in density of occluding radial lengths (i.e., linear paths) elevated access. The implications of this result should find footing with urban and transportation planners focused on promoting food access by enhancing the sight lines of bicycling or walking corridors.

This study has limitations that we should note. First, the sample size was small, and this constrains the generalizability of the study. Secondly, despite the complexity of this analysis, it is unknown how food access is affected by changes in ethnic food store density over time. A longitudinal analysis could be undertaken in future research to solve this problem. Additionally, we treated ethnic food access as a geographic problem, which does not tell the whole story. For instance, we acknowledge that we did not collect food basket information, menu data, business hours, time-sensitive individual shopping behaviors, or food preferences. These factors influence objective and perceived food outlet access and should be considered in future research. Lastly, our research included reasonable active travel distances in our accessibility index; however, we did not collect data on preferred travel modes. This information would provide deeper insights into the feasibility of reaching the food outlets.

This research examined an often-neglected food resource, ethnic food outlets. To more fully understand their potential for mitigating food insecurity, we analyzed two Michigan cities by using an ESDA and geospatial modeling approach. Our study identified important information about ethnic food stores that have not been reported elsewhere. Our findings indicate that urban planners should incorporate ethnic food stores into attempts to bring healthy foods to inner city neighborhoods.

## Figures and Tables

**Table 1:** Demographic and socioeconomic statistics.

Demographic Characteristics	Flint	Grand Rapids
Population	107,824	189,800
Median age	34	33
Median income	$29,260	$41,027
Post high-school diploma	49.60%	49.85%
Population under 18	28.43%	25.67%
Population change, 2000–2010	−15.90%	−2.73%
Mean family size	3.0	3.2

Source: U.S. Census Bureau, 2010.

**Table 2:** Ethnic food outlets in each city.

Food Outlets	Flint		Grand Rapids	
Type	*n*	Examples	*n*	Examples
Restaurant	57	Anacelia’s, Wah Nam Restaurant	141	Fazoli’s, Foo Yen, El Sol Azteca
Grocer	9	Bam’s African Market, Farah Khouri Supermarket	22	Quis Qoella, Sakim Grocery, Spice of India

**Table 3:** Variable descriptions.

Variables	Description
Dependent variable	
RAI	Revealed accessibility index, transformed using the square root
Independent variables	
EFO	Ethnic food outlet type (dummy, restaurant = 1, grocer = 0)
Demographics	
Male	Density of males per km
Population	Population density (persons per km)
Low wage workers	Number of workers per km earning $1250 per month or less
3 or more races	Density (per km) of persons identified as three or more races
Race-white	Density (per km) of persons of one race: white
High school diploma	Population density (per km) of those with a high school diploma
Environment	
Crime	Quantity of all crimes per km
Pedestrian intersections	Pedestrian-oriented intersection (3 or 4) density
Multi-modal intersections	Intersection density in terms of multi-modal intersections having three legs per square mile
Roads	Auto-orientated road network density per km
Road intersections	Intersection density in terms of auto-oriented intersections per square mile
Parks	Park area density per km
Urban morphology	
Shortest line distance	Mean metric shortest length pathways from each point to every other point per area
Shortest path angularity	Mean number of angular deviations per zone
Isovist area	Total viewable area from any space in the system
Entropy	Mean measure of the physical order of the system
Visual depth	The mean number of steps from any space to any other space in the system
Visual control	Mean value of visually dominant areas, high values = high visual dominance
Occlusivity	Mean measure of optic discontinuity in an environment. High values indicate long lengths (optic continuity) of occluding radials

**Table 4:** Moran’s *I* test of revealed accessibility index (RAI) in each city’s active living zone.

Statistics	Grand Rapids			Flint		
	Walking	Mass-transit	Bicycling	Walking	Mass-transit	Bicycling
Moran’s *I*	0.140	0.082	0.128	0.325	0.787	0.327
Expected *I*	−0.006	−0.006	−0.006	−0.016	−0.018	−0.016
*z*-score	0.684	0.415	0.614	0.857	1.946	0.846
*p*-value	0.493	0.678	0.538	0.391	0.051 *	0.397

* *p* < 0.1.

**Table 5:** Flint (*n* = 60) and Grand Rapids (*n* = 161) walking zone fully adjusted model coefficients and diagnostic outputs.

Statistical Approach	OLS				SAR				GWR			
Variables	Grand Rapids		Flint		Grand Rapids		Flint		Grand Rapids		Flint	
Independent Variables	Estimate	SE	Estimate	SE	Estimate	SE	Estimate	SE	Min	Max	Min	Max
EFO ^a^	−0.219 **	4.503	-	-	−8.849 **	6.948	-	-	−14.039	−4.148	4.265	5.368
**SES**												
Population	-	-	-	-	-	-	-	-	0.000	0.004	−0.011	−0.005
Low wage workers	-	-	-	-	-	-	-	-	−0.046	−0.005	0.010	0.074
**Environment**												
Crime	-	-	−0.258 *	0.033	-	-	-	-	−0.000	0.002	−0.057	−0.049
Pedestrian intersections	-	-	0.461 ***	1.202	-	-	0.579 ***	1.337	−0.444	0.935	0.152	0.473
Roads	0.148 *	9.407	−	−	0.13 *	8.707	-	-	−2.064	41.268	−13.023	−7.074
**Urban Morphology**												
Shortest line distance	-	-	-	-	-	-	0.45 **	0.003	0.000	0.001	0.001	0.002
Isovist area	-	-	−0.214 *	<0.001	-	-	−0.33 **	0.175	0.000	0.000	−0.000	−0.000
**Fit Statistics**												
*R*^2^	0.075		0.277		0.172		0.513		0.210		0.193	
Adj. *R*^2^	0.033		0.175		-		-		0.091		0.012	
AICc	1336.024		552.32		1321.609		533.37		1328.198		562.48	
rho	-		-		0.8		0.8		-		-	
Alpha	-		-		1.0		1.0		-		-	
Moran’s *I* ^b^	0.121		0.136		0.121		0.135		0.141		0.141	

^a^ reference—grocer (0); ^b^ Spatial relationships conceptualized using inverse Euclidian distance; * *p* < 0.1; ** *p* < 0.05; *** *p* < 0.01; VIF index values for all covariates < 10.0; -, no statistically significant relationship.

**Table 6:** Flint (*n* = 56) and Grand Rapids (*n* = 159) mass-transit zone fully adjusted model coefficients and diagnostic outputs.

Statistical Approach	OLS				SAR				GWR			
Variables	Grand Rapids		Flint		Grand Rapids		Flint		Grand Rapids		Flint	
Individual Variables	Estimate	SE	Estimate	SE	Estimate	SE	Estimate	SE	Min	Max	Min	Max
EFO ^a^	−0.837 ***	0.013	−	−	−0.852 ***	0.013	-	-	−0.330	−0.191	−0.018	0.007
**Demographics**												
3 or more races	-	-	0.292 **	0.006	-	-	-	-	−0.005	0.001	0.006	0.030
Male	−0.750 ***	<0.001	−0.835 ***	<0.001	−0.740 ***	<0.001	−0.028 ***	0.02	−0.000	−0.000	−0.002	−0.001
**Environment**												
Road intersections	-	-	-	-	-	-	-	-	−0.102	0.041	−2.567	1.144
Multimodal intersections	0.307 **	0.009	0.344 ***	0.031	0.236 **	0.009	0.291 **	0.054	0.003	0.046	0.084	0.221
**Urban Morphology**												
Visual Depth	-	-	0.387 **	0.047	-	-	-	-	−0.009	−0.004	−0.012	0.196
Shortest path angularity	-	-	−0.425 ***	0.019	-	-	−0.361 *	0.032	−0.004	0.000	−0.102	−0.025
**Fit Statistics**												
*R*^2^	0.814		0.883		0.907		0.908		0.885		0.938	
Adj. *R*^2^	0.807		0.876		-		-		0.870		0.911	
AICc	−494.041		−203.588		−600.332		−209.345		−555.407		−216.789	
rho	-		-		0.600		0.900		-		-	
Alpha	-		-		1.0		1.1		-		-	
Moran’s *I* ^b^	−0.168		0.064		−0.050		0.555		−0.272		−0.376	

^a^ reference—grocer (0); ^b^ Spatial relationships conceptualized using inverse Euclidian distance; * *p* < 0.1; ** *p* < 0.05; *** *p* < 0.01; VIF index values for all covariates < 10.0; -, no statistically significant relationship.

**Table 7:** Flint (*n* = 60) and Grand Rapids (*n* = 161) bicycling zone fully adjusted model coefficients and diagnostic outputs.

Statistical Approach	OLS				SAR				GWR			
Variables	Grand Rapids		Flint		Grand Rapids		Flint		Grand Rapids		Flint	
Individual Variables	Estimate	SE	Estimate	SE	Estimate	SE	Estimate	SE	Min	Max	Min	Max
EFO ^a^	−0.711 ***	0.019	−0.110 *	0.005	−0.678 ***	0.019	−0.100 *	0.004	−0.173	−0.166	−0.010	−0.006
**Demographics**												
Race: white	−0.408 ***	<0.001	−0.877 ***	<0.001	−0.376 **	<0.001	−0.871 ***	<0.001	−0.000	−0.000	−0.000	−0.000
High school diploma	-	-	0.169 **	<0.001	-	-	0.156 **	<0.001	0.000	0.000	0.000	0.000
**Environment**												
Parks	-	-	0.229 **	0.036	-	-	0.232 **	0.038	0.000	0.000	0.101	0.106
**Urban Morphology**												
Visual Control	-	-	−	−	-	-	-	-	2.133	4.397	−1.237	−0.298
Occlusivity	-	-	0.899 ***	<0.001	-	-	0.875 ***	<0.001	−0.000	0.000	0.000	0.000
**Fit Statistics**												
*R*^2^	0.496		0.841		0.530		0.833		0.506		0.852	
Adj. *R*^2^	0.480		0.819		-		-		0.475		0.826	
AICc	−431.607		−348.905		−439.415		−342.003		−431.734		−350.499	
rho	-		-		0.8		0.3		-		-	
Alpha	-		-		1		1.1		-		-	
Moran’s *I ^b^*	0.179		0.528		0.206		0.618		0.176		0.558	

^a^ reference—grocer (0); ^b^ Spatial relationships conceptualized using inverse Euclidian distance; * *p* < 0.1; ** *p* < 0.05; *** *p* < 0.01; VIF index values for all covariates < 10.0; -, no statistically significant relationship.
